# Self consistency grouping: a stringent clustering method

**DOI:** 10.1186/1471-2105-13-S13-S3

**Published:** 2012-08-24

**Authors:** Bong-Hyun Kim, Bhadrachalam Chitturi, Nick V Grishin

**Affiliations:** 1 Biochemistry Department UT Southwestern Medical Center, Dallas, TX, USA; 2 Howard Hughes Medical Institute, UT Southwestern Medical Center, Dallas, TX, USA; 3 Department of Computer Science Amrita Vishwa Vidyapeetham University, Amritapuri Campus, Kerala, India; 4 School of Biotechnology, Amrita Vishwa Vidyapeetham University, Amritapuri Campus, Kerala, India

## Abstract

**Background:**

Numerous types of clustering like single linkage and K-means have been widely studied and applied to a variety of scientific problems. However, the existing methods are not readily applicable for the problems that demand high stringency.

**Methods:**

Our method, self consistency grouping, i.e. *SCG*, yields clusters whose members are closer in rank to each other than to any member outside the cluster. We do not define a distance metric; we use the best known distance metric and presume that it measures the correct distance. SCG does not impose any restriction on the size or the number of the clusters that it finds. The boundaries of clusters are determined by the inconsistencies in the ranks. In addition to the direct implementation that finds the complete structure of the (sub)clusters we implemented two faster versions. The fastest version is guaranteed to find only the clusters that are not subclusters of any other clusters and the other version yields the same output as the direct implementation but does so more efficiently.

**Results:**

Our tests have demonstrated that SCG yields very few false positives. This was accomplished by introducing errors in the distance measurement. Clustering of protein domain representatives by structural similarity showed that SCG could recover homologous groups with high precision.

**Conclusions:**

SCG has potential for finding biological relationships under stringent conditions.

## Background

Grouping related objects into clusters has been one of the most widely used tools in many disciplines including biological sciences [1]. Typically, clustering methods endeavor to form clusters of objects where the objects within the cluster are more similar than the objects outside the cluster. They either partition the data set into groups or agglomerate the single objects into groups. Most methods require some knowledge; *e*.*g*. the number of clusters *K* in *K*-means or some kind of similarity measure cut-off (distance cut-offs in single linkage) [2,3]. In general, for biological applications the number of (sub)clusters to be formed (*e.g*. the number of homologous groups in protein space), or the cluster size limit to determine the boundaries is not known. Moreover, specificity is crucial, *i.e*. clustering biologically distant objects is worse than not clustering them [4].

We developed a method that we call *self-consistency grouping i.e. SCG*, that directly uses the natural definition of clustering objects, *i.e.* the objects in the cluster are more similar to each other than to the objects outside of the cluster [1]. We define the asymmetric *distance* between an object *x* and an object *y* as *p* if there are *p*-1 objects (including itself) that are more similar to *x* than *y*. A set of *k* objects form a *cluster* if the rank of any object within the cluster with respect to any other in the cluster is at most *k*. SCG can be considered as an agglomerative hierarchical clustering method since it starts to form clusters with individual objects and forms larger clusters until no more clusters can be formed. It can also be considered as a generalized version of the method used in building Clusters of Orthologous Groups (COG) database [5]. In building the COG database, the authors used a list of mutually most similar proteins pairs whereas we employ X-mutually nearest neighbors, where X is not specified and is solely dependent on the input data. Mutual nearest neighbors idea was introduced in 1973 by Jarvis and Patrick [6], then employed by others [7-9]. Nagendraswamy and Guru [8] suggested a similar consistency idea which is more relaxed compared to SCG, in the K-mutual nearest neighbors context.

## Methods

### Terminology

A set of *k* objects form a *cluster* if the rank of any object *y* with respect to any other *x* in the cluster is at most *k*; *i.e*. *rank*(*x*,*y*)≤*k*. This stringent criterion yields very low false positives. The cluster definition is symmetric whereas the distance metric (the rank of similarity) is asymmetric. SCG partitions the dataset and generates a hierarchy within each partition.

The total number of objects in the data set is *n*. We disallow a cluster of size *n*. *C_s_* is a *subcluster* of a cluster *C* if objects of cluster *C_s_* are a proper subset of objects of *C*. A cluster can subsequently become a subcluster of another cluster. An *independent cluster* is a cluster that is not a subcluster of any other cluster. However, it may have subclusters of its own. For example, consider the following grouping produced by SCG on seven objects {a,b,c,d,e,f,g}: (a, ((b,c) d)) (e,f,g,h). It has two independent clusters, (a, ((b,c) d)) and (e,f,g,h); (b,c) is a subcluster of ((b,c) d). Likewise, ((b,c) d)) is a subcluster of (a, ((b,c) d)). We call the specification of subclusters within a cluster as *structure*. An algorithm specifies the *complete structure* if it identifies all subclusters.

We call an algorithm *deterministic* if the numbering of the objects does not alter the output, otherwise it is *non-deterministic*. The *candidate index of a* given a set *S* is defined as the maximum value of *rank*(*x*,*y*) ∀ {*x*,*y*} ∈ *S*.

The input consists of a rank matrix, *rmatrix*, where *rmatrix*[*i*,*j*] has *rank*(*i*,*j*). Hence, *rmatrix* consists of integers in (1,…,*n*) and each of its row is a permutation of (1,2,…,*n*). Each object is most similar to itself. Thus, the *rmatrix*[*i*,*i*]=1. The objective is to find the hierarchical structure of clusters where each cluster’s members are more similar to each other than to the members of the other clusters. Likewise, the members of a subcluster are more similar to each other than to the members of the other subclusters within a given cluster. For ease of computation, sorted rank matrix, *smatrix* is created from *rmatrix*. The entity *smatrix*[*i*,*j*] = *k* if *rank* [*i*, *k*] =*j*. Every object is most similar to itself, thus, *smatrix*[*i*,1] =*i*.

### Algorithms

We present three algorithms: *A*1, *A*2, and *A*3. *A*1 and *A*3 are equivalent, they are deterministic and specify complete structure whereas *A*2 does not specify the complete structure and it is non-deterministic. When comparing to other methods we call *A*2 as *SCG-fast*, SCG-fast is used because we compare the independent clusters of SCG to that of other methods.

Each of *A*1, *A*2, and *A*3 determines if the first *k* objects in row *o* (corresponding to object *o*) of *smatrix* form a cluster. They differ in the way *o* and *k* are chosen, *i.e*., they differ in the order of examining the objects (*i*.*e*. how *o* is chosen) and the manner in which the size of the next feasible cluster including *o* is chosen.

*A*1 is the most straightforward realization of SCG. *A*2 and *A*3 use *candidate indices* to improve efficiency. We make a key observation about the order in which objects must be explored based on their respective candidate indices. This observation yields *A*3.

Asymptotic worst case time complexity of all algorithms is the same. In practice, the fastest to the slowest algorithms in sequence are (*A*2, *A*3, *A*1). In practice, running *A*2 to find all independent clusters, followed by running *A*3 on each of the independent clusters was the fastest. *A*2 quickly finds all the independent clusters and in due process breaks down the original set of objects into several subsets. *A*3 runs faster on all the subsets combined compared to the original set.

#### Subcluster management

We use a tree data structure to manage subcluster structure. Initially every node representing an object is a root. We call roots (either singletons or that have children) as *tree objects*. When two or more tree objects *O*_1_…*O*_i_ form a cluster *C*, a new root *r* is created and all the roots denoting *O*_1_…*O*_i_ become children of *r*. From now on the *r* represents *C*. When one is examining an object *o*, if the first *k* elements in row *o* of *rmatrix* form a cluster *C*_1_ and if the first *k*+*p* elements form a cluster *C*_2_ then the additional *p* elements in *C*_2_ (w.r.t. *C*_1_) may form a subcluster. To determine this, we check to see if all of these elements have a common root *r_c_*. If so then we link *r_c_* to the root of *C*_2_. Hence, this method is efficient and does not increase the overall time complexity.

#### A1 algorithm

At the start of an iteration, all the objects are marked as valid for that iteration. For every valid object *o*, *A*1 checks if the first *f* objects in row *o* of *rmatrix* form a cluster. The variable *f* is initialized to two. It is incremented by one after each iteration. If a cluster is formed, it is stored as a root of the new tree that is formed from the individual nodes (representing objects or trees in case there are subclusters) as immediate children and all the objects in the cluster are marked as invalid for the current iteration. If larger clusters are formed in the subsequent iterations that include the existing clusters, then the existing clusters become subclusters of the newly formed cluster and the trees are merged accordingly to reflect the structure.

The algorithm examines all clusters from the smallest to the largest size and hence finds all clusters and their subclusters. Only clusters that contain invalid objects (hence already examined) are not examined. In *O*(*k*^2^) one can check if *k* objects form cluster. In the worst case, each object consumes ∑*_k_*_=2,_*_n_*_-1 _*O*(*k*^2^) = *O*(*n*^3^), a total of *O*(*n*^4^) for all the *n* objects.

#### A2 algorithm

For every object *o*, *A*2 checks if the first *o^i^* objects of *o^th^* row of *rmatrix* form a cluster. The variable *o^i^* is the index variable for object *o*. It is initialized to two. If the set of first *o^i^* objects do not form a cluster then index variable *o^i^* is set to the *candidate index* (defined earlier) of the first *o^i^* objects. If a cluster is formed then it is stored in a tree structure and *o^i^* is incremented by one. We call the first candidate index number of objects of the *o^th^* row as a *candidate cluster* for object *o*. When larger clusters are formed in the subsequent iterations, the structure is updated accordingly. This process is repeated until an invalid item is encountered or the end of the row is reached. At this time, the largest possible cluster containing this object has formed and all its members are marked as invalid. The same process is repeated at the next valid object. The computation terminates when no valid object remains. An object *o* is marked as invalid if it belongs to an independent cluster, which can be a singleton, *i*.*e*. {*o*}.

Let the first two objects in the *j^th^* row of *rmatrix* be {*j*,*m*}. The first object will be *j* itself because it is most similar to itself. Let the *rank*[*m*,*j*]= *r* (the score is asymmetric). There are two cases, either *r* =2 or *r*>2. In the first case we have a cluster of size two. Otherwise, the candidate index is *r*. If a candidate cluster does not form a cluster then the minimum size of the next candidate cluster is equal to the candidate index of the current candidate cluster. Similar to *A*1 the complexity is *O*(*n*^4^). However, in practice, the average number of candidate clusters for *A*2 is much lower than *A*1.

We note that when *A*2 examines an object *o* it considers all candidate clusters of *o* which includes the independent cluster that contains *o*. Due to the symmetry in the definition of SCG, a given object belongs to only one independent cluster. If an object *x* belongs to two clusters *C*_1_ and *C*_2_ then either *C*_1_⊂ *C*_2_ or *C*_2_⊂ *C*_1_. This is true because all the members of the smaller cluster have smaller ranks with respect to *o* and hence will be included in any larger cluster. Consider a cluster *C*=(1(2,(3,4)5)). When the algorithm examines object 1 it finds (1,2,3,4,5) where the structure of *C* is not known. Whereas if *C*=((((1,2), 3),4)5) then algorithm will find entire structure when it examines object 1. Thus, the complete structure is not always identified and depends on the ids. assigned to the objects. Thus, *A*2 is non deterministic.

**Lemma 1. ***A*2 finds all independent clusters.

**Proof.** We prove this by contradiction. Let *C* be an independent cluster that is not identified by *A*2. Let the object with the smallest id. in *C* be *x*. After *A*2 examines all objects whose ids. are less than *x*, either *x* is valid or it is invalid. If *x* was invalid then *x* must be a member of a cluster *C*’ formed when an object with id. *y* (<*x*) is examined. This contradicts that *x* has the smallest id.. If *x* was valid then the algorithm will find the largest cluster involving *x* (which can be a singleton, *i.e*. *x* by itself). Note that *A*2 tries all candidate indices of *x*. This contradicts that *C* is not identifed. Thus, *A*2 finds all independent clusters.

#### A3 algorithm

*A*3 captures the computational advantage of not examining infeasible candidate clusters. For any given object *o* its index *o^i^* is initialized to two. For every valid object o, whose index is equal to the minimum value among all indices, *i.e*. *minimum index*, we check if the first *o^i^* objects of *o^th^* row of *smatrix* form a cluster. At the start of an iteration if no valid object remains the computation terminates. If a cluster is formed, it is stored in a tree structure. The indices of all objects in the cluster are incremented by one and these objects are marked as invalid for the current iteration. Similar to *A*2, if the first *o^i^* objects of *o^th^* row do not form a cluster then the index of *o* is set to the candidate index. If the index of any object equals *n* then that object is marked as invalid for the rest of the computation. When larger clusters are formed, they are handled as in *A*1 and *A*2.

*A*3 identifies all independent clusters. The following lemma proves that *A*3 also identifies all subclusters within a given independent cluster. Thus, similar to *A*1, it finds the entire subcluster structure.

**Lemma 2. ***A*3 finds all subclusters of an independent cluster C.

**Proof.** We prove this by contradiction. Let *S* be a subcluster of *C* that is not found by *A*3. Let |*S*| =*s* (< |*C*|) and the object with the smallest id. in *S* be *x*. Consider the execution of *A*3 when the minimum index equals *s*. When algorithm examines object *x*, it identifies the cluster *S*. Let the object with the smallest id. in *C* be *y*. Either *x*=*y* or *y*<*x*. In the former case *S* is identified when examining object *x* and in the latter case *S* should already have been identified when *y* was examined. In either case, when *C* is formed, *S* already exists; so, it is incorporated into *C*. This is a contradiction. Thus, *A*3 finds all subclusters.

#### Execution Trace

**Figure 1:**
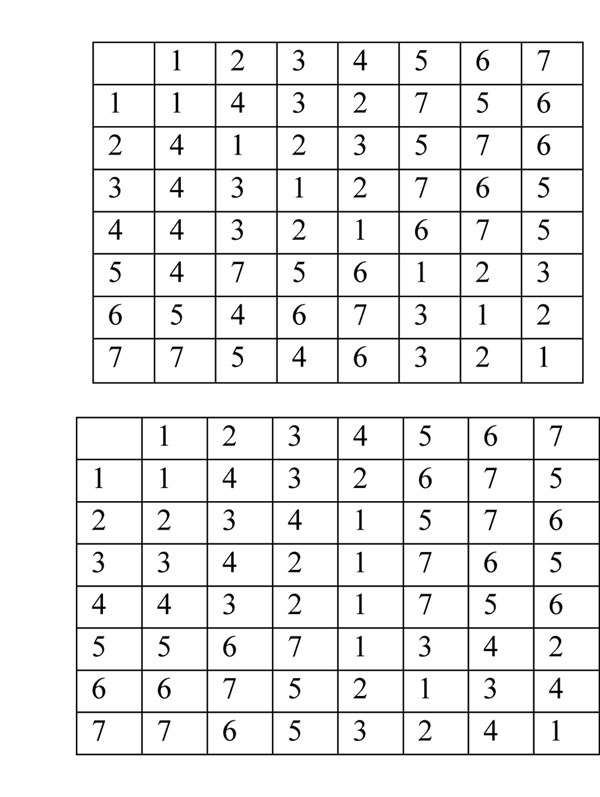
**Execution traces for SCG algorithms.** The two matrices *rmatrix* (upper matrix) and *smatrix* (lower matrix) are shown for a dataset of seven objects. We trace *A*1, *A*2 and *A*3 on this input. Note that the ranks are asymmetric.

Consider *rmatrix* and *smatrix* for seven objects (Fig 1.), where rows represent an object. In *rmatrix* the columns represent objects and the entry is the rank of the object denoted by the column number with respect to the object represented by the row, whereas, in the *smatrix* the column indicates the rank and the entry is the corresponding object id.

We trace all three algorithms on these matrices. *A*1 examines five potential clusters of size two, three clusters of size three, four clusters of size four and seven each of sizes five through seven and identifies: ( (1,2,(3,4)) (5,(6,7)) )

*A*2 finds {1,2,3,4} and {5,6,7} but subclusters are not known. *A*2 first examines {1,4} and *rank*(4,1)=4 so, the index is set to four. This results in identifying the cluster {1,2,3,4} and setting the index to five. The *rank*(6,4)=7; thus, 1,2,3,and 4 are marked as invalid and the next object i.e. 5 is considered. The first two objects, 5and 6 do not form a cluster and the *rank*(6,5) = 3. Thus, index of 5 is set to three and the first three objects 5,6, and 7 are checked. They form a cluster; so, the index is incremented to four. The fourth object is 1 and the *rank*(1,5) =7. The objects 5,6, and 7 are set to invalid and the computation halts because no valid objects remain. In total, *A*2 examines four potential clusters, two of size two, one each of sizes three and four.

*A*3 examines five potential clusters of size two, two of size three and one of size four. In *A*3, all indices are initialized to two and objects are checked to determine if they form clusters of size two. The objects 4 and 7 form clusters of size two with 3 and 6 respectively. For the objects that form a cluster the indices are incremented by one and for the rest the indices are set to their corresponding candidate indices. Thus we have indices of 1,2,3,4,5,6, and 7 as 4,3,3,3,3,3, and 3 respectively. In the next iteration, only objects with minimum index, *i.e.* with a value of 3 are considered. The first such object is 2 and it forms {2,3,4}, a cluster of size three. Thus, 2, 3, and 4 are invalid for the current iteration. The next object is 5 and it forms {5,6,7}, a cluster of size three. The indices of objects 2-7 are set to four and index of object 1 has a value of four from earlier iteration. In the next iteration the minimum index is four (3+1). The first object examined, 1, forms the cluster {1,2,3,4} of size four. Thus, 1, 2, 3, and 4 are marked as invalid (for the current iteration) and their indices are all set to five. The remaining objects, 5-7, which also have an index of four do not form any clusters, in particular, when we examine the first four entries for these objects (*i*.*e*. 5-7) in *rmatrix* we find that *rank*(1,5) = 7, *rank*(2,6) = 7, in and *rank*(3,5) = 7. Thus, objects 5,6, and 7 are removed from the valid list and will not be considered for the rest of the computation. We have four valid objects 1-4 all with indices of five. Examining the first five objects in the rows 1-4 of *rmatrix* corresponding to objects 1-4 for determining their candidate indices, we note the following. In first row *rank*(1,2) =4 and *rank*(1,6) =5; however *rank*(2,6) =7. In the second row *rank*(2,5)=5 but *rank*(5,2)=7. In the third row *rank*(1,7) =6 but *rank*(7,1) =7. In the fourth row *rank*(4,1) =4 and *rank*(4,7) =5, however, *rank*(7,1) =7. Thus, objects 1-4 all have the same candidate index of *n* (*i*.*e*. seven). Thus, they are removed from the valid list. The computation terminates because there are no valid objects left.

#### Execution time comparisons of A1, A2, and A3

**Figure 2:**
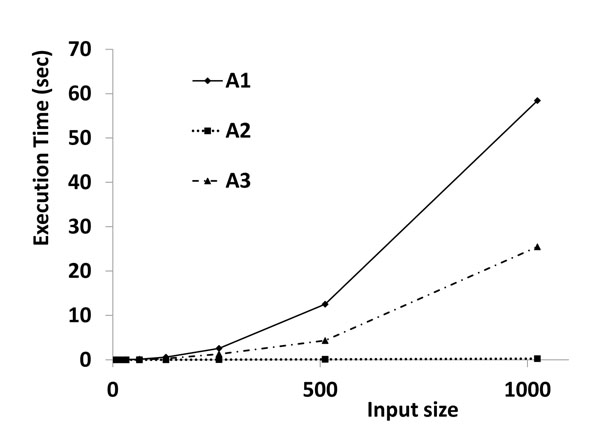
**Comparisons of execution times of SCG algorithms**. Comparison of execution times of SCG algorithms. *A*1 is the slowest followed by *A*3 and *A*2. Note that *A*2 is guaranteed to identify the independent clusters and might not identify the complete structure.

The three algorithms described above (*A*1, *A*2 and *A*3) were implemented in python (ver. 2.3); the code will provided upon request. The implemented code along with tools for clustering analysis and data files used for this study can be found at http://prodata.swmed.edu/scg/. For this implementation, we measured the execution time for *A*1, *A*2 and *A*3 (Table 1 and Fig 2). The data set at each size is created by generating random points in 2D. Euclidian distance is the distance measure. As expected, *A*1 was the slowest followed by *A*3 and *A*2 respectively. *A*2, which is not guaranteed to find the complete structure, is the fastest. The execution time *t*(*n*) for the input size *n* is θ(*n*^2^) for *A*1 and *A*3. For *A*1, *t*(*n*) = 6E-05*n*^2^ - 0.0077*n* + 0.250, R^2^ the coefficient of determination is 0.9999. However, for *A*2, *t*(*n*) = 0.0003*n* - 0.009, R² = 0.9929.

**Table 1:** Execution time measurements of SCG algorithms

	A1	A2	A3
8	0.0011	0.00055	0.0009
16	0.0061	0.0025	0.0042
32	0.025	0.0039	0.01
64	0.131	0.009	0.051
128	0.594	0.016	0.276
256	2.584	0.049	1.295
512	12.54	0.118	4.34
1024	58.429	0.27	25.47

## Results

We compare SCG to well-known agglomerative clustering algorithms: complete linkage (CL), single linkage (SL), and average linkage (AL). We compared the methods with simulated data and with SCOP datasets. Among these methods, CL is the most similar method to SCG.

CL clusters a pair of objects if the distance between them is less than a specified cut-off. CL merges a pair of clusters if the maximum distance between any pair of objects where the first object is from the first cluster and the second object is from the second cluster is at most the chosen cut-off. Thus, it is stringent akin to SCG.

In contrast to CL, SL a popular method, groups the objects aggressively. When forming hierarchies of clusters SL uses the minimum distance between clusters. AL is another popular method that balances the approaches of CL (very conservative) and SL (very aggressive). Thus, CL, AL and SL capture a wide gamut of behavior and comparing SCG to them will yield a fair assessment of SCG.

SCG uses the rank matrix (that is obtained from a distance metric) and inherent consistency in the ranks whereas CL directly uses distance matrix along with an explicit parameter (cut-off). Let the distance cut-off for CL be δ. We note that SCG can yield a (sub)cluster in which the maximum distance between a pair objects is greater than δ. Let *A* be subcluster of cluster *B* in SCG. The maximum distance between *o*_1_ and *o*_2_ can be greater than δ where *o*_1_, *o*_2_ are objects of *B* where they can both be members of *A*. Thus, CL appears to be more stringent than SCG. It will not cluster a set of objects if the maximum distance between any pair is greater than δ. SCG has no such artificial restriction. In contrast to the above scenario, consider a scenario where two clusters *A* and *B* are to be grouped by CL and SCG. If the distance between any pair *a*, *b* where *a* ∈ *A* , *b* ∈ *B* is at most δ then CL groups them together. However, SCG will not do so if the *rank*(*a*,*b*) (or *rank*(*b*,*a*)) > |*A*|+*|B|* for any *a*, *b*. Thus, SCG and CL are stringent in different ways. This renders their comparison interesting.

After SCG produces the clusters, one can rank the clusters by the ascending value of the maximum distance between any pair of objects within a cluster. This will rank the natural clusters in the data from the best (first) to the worst (last).

We perform a balanced comparison of clustering methods by measuring the number of clusters and the number of incorrect pairs. If the goal of clustering is to find robust clusters, AL is the most suitable. However, if the goal is to minimize false positives in the presence of large number of random errors, SCG is a good candidate. This suits many problems in biological sciences; *e.g.* genome-wide associations or detecting homologous proteins. In such problems, biologists are keener on eliminating the false positives (incorrect pairs) than avoiding false negatives (missing pairs). This probably stems from the fact that each linkage might form a new hypothesis that needs to be tested. False positives can waste time and resources. Moreover, a priori cut-off score or cluster size are not known for many biological problems. Thus, SCG is a good candidate for these problems.

### Comparison of methods on simulated data

**Figure 3:**
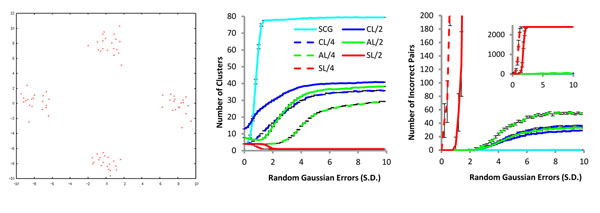
**Effect of random errors introduced in distance measurements**. (a) Dataset: We randomly generated a dataset of 80 points around four centers (0,8), (0, -8), (8,0) and (-8,0), 20 points for each center. Each point was offset from the center in both X and Y directions by a random amount following normal distribution (µ=0 and SD= 1). (b) Effect of random error on average cluster sizes: For the given dataset of 80 points, the Euclidean distances were calculated. Then we perturbed each pairwise distances with random value following a Gaussian distribution with µ=0 and SD shown on X axis. Note that SD = 0 implies that there are no perturbations. These distances are used to build clusters using SCG (cyan line), complete linkage (CL, blue line), average linkage (AL, green line) and single linkage (SL, red line). Since CL, AL and SL requires score cut-offs, we measured the clustering with distance cut-off values of 2 (conservative), and 4 (less conservative) denoted by the numbers following “/” (solid lines and dotted lines respectively). Finally, the number of clusters was measured per method per cut-off (this includes singletons). Thus, the maximum value can be 80 (all singletons) and the minimum possible value is 1. The ideal number is 4 by design (Fig 3. (a) ). The error bars shown at different points of the curves (each representing a method) are derived from 100 perturbations for a given SD. Note that the SCG shows steepest rise in the number of clusters. (c) Effect of random error on cluster qualities: Legends and the unit of X-axis are same as in (b). After each method identifies clusters, we enumerate all pairs within a cluster, *e.g*. the cluster {1,2,3} is decomposed into three pairs 1-2, 1-3, and 2-3. If both objects of any pair do not belong to the same reference cluster then we increment the number of incorrect pairs by one. Note that the number of incorrect pairs is intrinsically related to the number of clusters. If the number of clusters is 1, meaning all objects are grouped into one cluster, the number of clusters is minimum and that is reflected in the big numbers of incorrect pairs (See SL/4). If the number of clusters is 80, (similar to SCG at high errors) then no incorrect pairs exist.

We compare SCG to other popular agglomerative clustering methods. We generated 100 random test datasets. As shown in Fig 3. (a), each dataset contains 80 points generated around 4 centers in 2D, 20 points around each center. We employed the Euclidean distance as the distance measure and computed it for all pairs to be used by SCG-fast (*A*2), complete linkage (CL), average linkage (AL), and single linkage (SL) clustering methods [10]. All methods except SCG used distance cut-offs of 2 (conservative) and 4 (less conservative) (Fig 3. (b) and (c) ). To simulate the effect of measurement errors of real world dataset, random Gaussian perturbations with mean, µ=0 and standard deviations (SD) shown in X axis (Fig 3. (b) and (c)) were introduced when the distances were computed. The larger SD is more likely to introduce larger errors. The plot of cluster size versus SD shows how sensitive a method is to errors.

Ideally the number of clusters should be 4, denoting 4 groups of points in the plane as shown in Fig 3. (a). If more (less) than 4 clusters are found then the objects are overly split (grouped). If no (all) objects are grouped, the number of clusters will be 80 (1). As we postulated, SCG is very sensitive to the errors. It correctly identifies 4 clusters when the errors are small (the cyan curve in Fig 3. (b) and (c). However, as bigger errors are introduced the number of clusters identified by SCG increase rapidly due to the inconsistencies in ranks. CL, which is also *stringent* like SCG, shows similar increase as errors become larger, but it is less sensitive than SCG (Fig 3). ). Note that the conservative CL/2 yields more number of clusters than SCG for smaller values of error. However, the growth rate of the clusters in CL/2 tapers off whereas the same for SCG is exponential until it plummets when the number of clusters is close to the maximum value ( corresponding to S.D. of the error ~ 1.5). Among all methods, AL at cut-off 4 (dotted green line in Fig 3) is the most robust. The number of clusters remains at 4 for bigger errors with very low incorrect pairs. SL is also very sensitive to the errors. When the error is larger, SL quickly groups all objects into one cluster (SD>2, red lines in Fig 3. (b)) and the number of incorrect pairs also becomes very high, as shown in the Fig 3. (c). Notably, apart from SCG, all other methods eventually made a few incorrect pairs.

### Comparison of methods in clustering protein structure

In general, protein structures were considered hard to cluster with conventional clustering methods without human intervention [4]. We classified protein structures with SCG-fast, CL, AL, and SL (similar to the comparison of the previous section). We selected 9528 representative protein domain structures at 40% sequence identity having all alpha, all beta, alpha/beta, and alpha+beta from Structural Classifications Of Proteins (SCOP) ver. 1.75 [11]. Then Z-scores were measured for all pairs (~50,000,000) of the structures among the selected SCOP domain structures with DALI [12], one of most widely used structural comparison program. The similarity scores measured by DALI can be found at http://prodata.swmed.edu/scg/dali/. SCG identified 4965 independent clusters. In contrast to the test based on simulated data of the previous section, we set the parameter that determines the number of clusters for all other methods to this value, 4965 (table 2), in order to compare different clustering methods without a bias.

**Table 2:** Comparisons of clusters built by different methods to the reference SCOP fold classification (Total # of domains clustered:9528)

	SCG	CL	AL	SL
Total number of clusters*	4965	4965	4965	4965
Number of non-singleton clusters	1926	1561	1263	975
Number of incorrect pairs	102	214	2952	6440
Percentage of incorrect pairs	(0.2)	(0.4)	(3.5)	(3.7)
Number of correct pairs	46938	50948	81280	166386
Percentage of correct pairs	(99.8)	(99.6)	(96.5)	(96.3)

*Total number of clusters was fixed at the number of clusters determined by SCG for a fair comparison of different methods.

**Figure 4:**
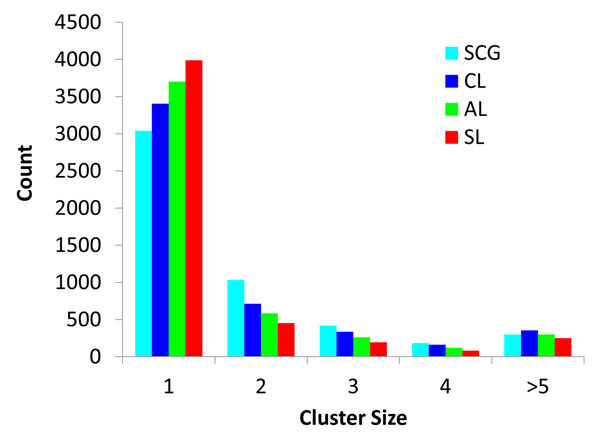
**SCG, CL, AL, and SL clustering results of SCOP domains based on structural similarity score**. The same color scheme was used as in Fig 3. The graph represents the cluster sizes (X axis) and the corresponding number of clusters (Y axis). This figure shows that all clustering methods yielded many small clusters or singletons and few big clusters. Note that the clusters with sizes greater than 5 are all lumped together.

The SCG clustering of SCOP domains shows that many of clusters are very small, ~1/3 of total protein domains form singleton clusters (3039 domains) and only few domains form relatively bigger clusters (see Fig 4). CL, AL and SL showed similar distributions, although CL shows the most similar cluster size distribution to SCG. SL shows the largest number of singletons. Among the three methods, CL is the most similar to SCG as shown in table 3. Compared to SCG other methods produce more singletons and lower numbers of clusters with sizes ranging from two to four. Clusters of protein structures constructed by these different methods were compared to SCOP folds built by experts and the number of correct and incorrect pairs were calculated same way as in Fig 3. (c). Similar to the simulation results (Fig 3. (c)), SL and AL show a larger percentage of incorrect pairs (3.7% and 3.5% respectively, see Table 2), whereas SCG and CL show only 0.2% and 0.4% incorrect pairs respectively. This reflects the fact that both SCG and CL are more conservative than others are.

**Table 3:** Similarities of clusters built by different methods

	SCG	CL	AL	SL
SCG	1.0	0.78	0.59	0.36
CL		1.0	0.74	0.44
AL			1.0	0.65
SL				1.0

F-measures are used for similarities between cluster similarities. F-measure is formally defined as a harmonic mean of precision and recall [13].

We would expect similar results to the clustering done on simulated data if the scores were perfect and the grouping was done objectively. Note that we used Euclidian distance in simulation (Fig 3. (a)), which is considered as a perfect measure, and SCG yielded no incorrect pairs. However, the DALI Z-score is a structural similarity score as measured by an approximate algorithm and the SCOP database is manually curated. Thus, one may reasonably expect that these two phenomena are not in perfect synchronization. We attribute the incorrect pairs found by SCG to the differences between the metric used for classification done by SCOP, *i*.*e*. human curation and the DALI Z-scores.

### SCG method in iteration

In some cases, for increasing the average cluster size, a few false positives can be tolerated. Here, the stringency of SCG becomes an issue. So, we designed iterative SCG or iSCG. The iteration is similar to other agglomerative methods. In the first iteration, SCG finds all independent clusters, subsequently each independent cluster is considered as an object. Then the rank matrix is updated for the next iteration, the new ranks can be determined based on one of the following strategies: i. most similar relationships, ii. average similarities between two groups, or iii. the most distant relationships, roughly corresponding to SL, AL, and CL, respectively. iSCG demonstrated correlation between the number of false positives and the number of iterations.

## Concluding remarks

We studied a clustering method that has no restriction on the size or the number of clusters. We designed two improvements *A*2 and *A*3 over the direct algorithm *A*1, and compared their execution times. *A*2 is guaranteed to identify all independent clusters (but not the complete structure); time wise, it is the most efficient. These independent clusters might suffice for some applications. If the complete structure is required, *e*.*g*. drawing dendrograms, *A*3 can be used.

Comparing SCG to other clustering methods demonstrated that SCG is very conservative. In simulation results, SCG did not yield any false positives. Because of its stringency, SCG can be used to validate the correctness of a distance metric in addition to clustering objects into groups. Moreover, SCG formed very accurate groups of protein structures indicating its potential applicability in exploring other biological data, such as microarray expression data, genome wide association studies etc.

## Competing interests

There are no competing interests.

## Authors' contributions

NVG conceived and supervised the study; BK and BC designed the algorithms; BK conducted the computational experiments analyzed the results; All authors participated in the preparation of this manuscript.

## Appendix

### Pseudocode
